# Etiology of severe acute respiratory infections among adults in northern Thailand using multiplex PCR: A post-COVID-19 surveillance study (2023–2024)

**DOI:** 10.1371/journal.pone.0350198

**Published:** 2026-06-09

**Authors:** Nang Kham-Kjing, Rathakarn Kawila, Patcharaporn Tariyo, Kittiyaporn Puapun, Sirinat Wongchotbrorirak, Nicole Ngo-Giang-Huong, Sayamon Hongjaisee, Woottichai Khamduang

**Affiliations:** 1 Department of Medical Technology, Faculty of Associated Medical Sciences, Chiang Mai University, Chiang Mai, Thailand; 2 LUCENT International Collaboration, Faculty of Associated Medical Sciences, Chiang Mai University, Chiang Mai, Thailand; 3 Nakornping Hospital, Chiang Mai, Thailand; 4 Maladies Infectieuses et Vecteurs: Écologie, Génétique, Évolution et Contrôle (MIVEGEC), Agropolis University Montpellier, Centre National de la Recherche Scientifique (CNRS), Institut de Recherche Pour le Développement (IRD), Montpellier, France; 5 LMI PRESTO, Faculty of Associated Medical Sciences, Chiang Mai University, Chiang Mai, Thailand; 6 Research Institute for Health Sciences, Chiang Mai University, Chiang Mai, Thailand; Defense Threat Reduction Agency, UNITED STATES OF AMERICA

## Abstract

**Introduction:**

The COVID-19 pandemic disrupted the circulation patterns of respiratory viruses. In tropical regions like Thailand, ongoing surveillance is essential to understand the etiology of severe acute respiratory infections (SARIs) in the post-pandemic era. We investigated the etiology of SARI among hospitalized adults in the post-pandemic era.

**Methods:**

We conducted a retrospective analysis of respiratory samples collected from adult patients (≥18 years) who were hospitalized at a regional hospital in Chiang Mai, Thailand, between November 2023 and April 2024 due to symptoms of SARI (fever of ≥38°C, cough, and onset within 10 days). Sputum and/or nasopharyngeal swab were collected at admission and tested using a multiplex real-time PCR assay targeting 22 respiratory viruses and *Mycoplasma pneumoniae* (Siemens Healthineers Fast Track Diagnostics and Tellgen SARS-CoV-2 Nucleic Acid Detection Kit). Demographic, clinical, treatment, and comorbidity data were extracted from hospital records, and descriptive statistics were used to summarize patient characteristics and pathogen distribution.

**Results:**

Among 101 hospitalized SARI patients (median age 62 years (interquartile range 43–71); 57 males), 47 (47%) tested positive for at least one respiratory pathogen. The most frequently detected viruses were adenovirus (17/101, 17%) and rhinovirus (13/101, 13%). Co-infections occurred in 9% (9/101) of cases. Seasonal trends showed peaks of influenza A and rhinovirus in January, while adenovirus and enterovirus circulated consistently throughout the study period.

**Conclusions:**

Nearly half of adult SARI cases were associated with viral pathogens. Other SARI etiologies could be due to bacterial or fungal infections not tested in our study. The high rate of empiric antibiotic use highlights the need for broader and rapid molecular diagnostics. Enhanced pathogen-specific surveillance is essential to guide evidence-based clinical management in the post-pandemic context.

## Introduction

Severe acute respiratory infections (SARIs) contribute to considerable morbidity and mortality across all age groups, especially among children under five, the elderly, and individuals with comorbidities [1,2]. Prior to the emergence of severe acute respiratory syndrome coronavirus 2 (SARS-CoV-2), SARIs accounted for over 4.2 million deaths annually, with more than 90% occurring in low- and middle-income countries [3], where access to diagnostics and effective treatment is constrained [4]. The COVID-19 pandemic further amplified this burden, resulting in over 7 million additional deaths globally [5].

A diverse array of pathogens, including bacteria, viruses, fungi, and parasites, can cause severe acute respiratory infections. The etiological landscape varies depending on geography and diagnostic capacity. Historically, bacterial infections have been the predominant cause of severe pneumonia [6,7] and are often treatable with broad-spectrum antibiotics. However, viral pathogens are now increasingly recognized as significant contributors [8,9]. The overlap in clinical presentations complicates the differentiation between viral and bacterial etiologies, necessitating laboratory confirmation for an accurate diagnosis.

Conventional diagnostic tools, such as culture-based methods, have limitations in detecting viral pathogens. This leads to diagnosis errors or delays in treatment [10]. This diagnostic uncertainty often results in the empirical use of antibiotics, which are ineffective against viral infections and contribute to antimicrobial resistance [11]. Advanced molecular assays enable rapid detection of pathogens, allowing appropriate treatment and containment strategies to be implemented.

The COVID-19 pandemic also disrupted the transmission dynamics and seasonal patterns of common respiratory viruses [12,13]. Public health measures implemented to control SARS-CoV-2, including masking, physical distancing, and travel restrictions, significantly reduced the circulation of other respiratory viruses. Most SARI surveillance data from Thailand and globally were collected prior to the pandemic [14–17], and it remains unclear whether the etiological profile of SARI has changed in the post-pandemic context. In this study, we aimed to determine the prevalence and etiological spectrum of respiratory pathogens, especially the viruses circulating among adults hospitalized with SARI in Chiang Mai, northern Thailand, during the post-COVID-19 pandemic era. These findings will inform future public health strategies and improve clinical management through pathogen-specific surveillance.

## Materials and methods

### Patients and sample collection

This study retrospectively analyzed data from adults (≥18 years) who were hospitalized at a regional hospital in Chiang Mai, Thailand, between November 2023 and April 2024 due to severe acute respiratory infections (SARI). Eligible cases met the World Health Organization (WHO) case definition for SARI: acute respiratory illness with a history of fever or measured temperature ≥38°C, cough, symptom onset within the preceding 10 days, and hospitalization. This study only included patients who were admitted for the first time during the study period. Corresponding respiratory specimens collected during routine care were used for pathogen testing. This study estimated the pathogen detection prevalence of 1% with a margin of error of 2%, resulting in a required sample size of at least 95. Based on this calculation, we targeted a minimum of 100 cases to ensure adequate precision in preliminary analyses.

### Demographic and clinical data

Demographic and clinical data, including age, gender, underlying medical conditions, clinical presentation, symptom duration, medications (antibiotics, antivirals), ventilation usage, hospitalization duration, and clinical outcomes, were extracted from hospital records.

### Sample processing and laboratory testing

Sputum samples were collected and pre-treated with an equal volume of 0.1% dithiothreitol (DTT) (Thermo Fisher Scientific, MA, USA) and incubated at room temperature to ensure homogenization. The nasopharyngeal swab underwent nucleic acid extraction without prior treatment. Total nucleic acid was extracted using the Nucleic Acid Extraction Kit (Zybio Inc., Chongqing, China) with the automatic extractor Auto-Pure 32A (Allsheng, Hangzhou, China), according to the manufacturer’s instructions. Then, extracted nucleic acids were tested qualitatively with the FTD™ Respiratory Pathogens 21 multiplex real-time reverse-transcription polymerase chain reaction (RT-PCR) assay (Fast Track Diagnostics, Siemens Healthineers, Esch-sur-Alzette, Luxembourg), capable of detecting 20 respiratory viruses and *Mycoplasma pneumoniae*. Such viruses include influenza A virus (IAV), influenza A virus H1N1 swine-lineage (IAV [H1N1] swl), influenza B virus (IBV), human rhinovirus (HRV), human coronaviruses ([HCoV] strains 229E, HKU1, OC43, NL63), human parainfluenza viruses (HPIV 1–4), human metapneumoviruses (HMPV) A and B, human bocavirus (HBoV), human respiratory syncytial viruses (HRSV) A and B, human parechovirus (HPeV), enterovirus (EV), and human adenovirus (HAdV). This multiplex PCR panel was selected because the primary objective of this study was to characterize common respiratory viral pathogens circulating among hospitalized adult SARI patients during the post-COVID-19 period. In addition to the multiplex PCR panel, qualitative severe acute respiratory syndrome coronavirus 2 (SARS-CoV-2) detection was performed using the Tellgen SARS-CoV-2 Nucleic Acid Detection Kit (Tellgen, Shanghai, China). All PCR results were interpreted following the manufacturer’s instructions. Co-infections were defined as the detection of two or more pathogens and were reported if each target met the positivity criteria.

### Data analysis

Descriptive statistics were used to summarize participant characteristics, including age, sex, clinical symptoms, comorbidities, and the distribution of respiratory pathogens. Categorical variables were reported as frequencies and percentages, while continuous variables were expressed as medians with interquartile ranges (IQR). Comparisons of clinical outcomes (hospital stay duration, pneumonia, and death) between mono-infected and co-infected patients were performed using the Fisher’s exact test. A *p*-value < 0.05 was considered statistically significant. To illustrate the relationship between detected pathogens and clinical outcomes, a Sankey diagram was constructed, showing the distribution of cases across outcome categories: not severe, requiring special care, bronchiolitis, pneumonia, and death. The width of each flow represents the relative proportion of cases within each category. All analyses and visualizations were performed using RStudio (version 4.4.2; RStudio, MA, USA).

### Ethics statement

This study was approved by the Ethics Committee of the Faculty of Associated Medical Sciences (Approval No. AMSEC-66EM-011) and the local Ethics Committee at the hospital (Approval No. 112/66). Archived clinical data and leftover specimens from routine diagnostics were accessed on 8 November 2023 for research purposes. All clinical data were anonymized to protect participant confidentiality, and samples were coded to prevent the identification of individual participants. The investigators had access only to coded data and specimens, without direct identifiers, throughout data collection and analysis. Samples used were leftover specimens from routine diagnostics, exempting the study from individual consent under Thai ethical regulations.

## Results

### Demographic and clinical characteristics

Between November 2023 and April 2024, 101 hospitalized adult SARI cases were included. The median age was 62 years (IQR: 43–71), with 45% aged ≥65 years (S1 Table). Of these, 56% were male. All patients exhibited more than one symptom. Common symptoms included fatigue (98%), dyspnea (80%), sore throat (40%), myalgia (27%), rhinorrhea and headache (21%), chills (20%), and diarrhea (13%). Less frequent symptoms included vomiting (5%) and hoarseness (1%). Comorbidities were present in 78% (79/101) of patients, primarily hypertension (39%), pulmonary disease (20%), cardiovascular disease (19%), kidney disease (18%), and diabetes (18%). Two or more comorbidities were present in 50% (50/101) of patients. All patients had received COVID-19 vaccination, while 73% had also received seasonal influenza vaccination within the past 2 years. Chest X-rays were abnormal in 52% (53/101) of patients, predominantly showing patchy infiltrates (29%), pleural effusion (11%), fibrosis (4%), and interstitial infiltrates (4%). Most patients had short hospital stays: 38% stayed 3–4 days and 37% for 5–6 days. Only 7% remained hospitalized for more than 10 days. Mechanical ventilation was required in 11% of patients. Ninety-five (94%) received antibiotic therapy, and two (2%) received antivirals. The most frequent clinical diagnosis was pneumonia (42%), and the mortality rate was 8% (Table 1).

**Table 1:** Demographic and clinical characteristics of adult cases with severe acute respiratory infection.

	Median (IQR) or *n (%)*
**Age**	62 (43–71)
**Age (years old)**	
18–30	14 (14)
31–64	41 (41)
≥65	46 (45)
**Sex:** male	57 (56)
**Symptoms** ^**a**^	
Fever	101 (100)
Cough	101 (100)
Fatigue	99 (98)
Dyspnea	81 (80)
Others^b^	71 (70)
**Comorbidity/Medical history** ^**c**^	79 (78)
Hypertension	39 (39)
Pulmonary disease	20 (20)
Cardiovascular disease	19 (19)
Kidney disease	18 (18)
Diabetes	18 (18)
Others^d^	25 (25)
**Vaccinations**	
Seasonal influenza vaccinated	74 (73)
SARS-CoV-2 vaccinated	101 (100)
**Abnormal Chest X-rays** ^**e**^	53 (52)
• Patchy infiltration	29 (29)
• Pleural effusion	11 (11)
• Interstitial infiltration	4 (4)
• Fibrosis	4 (4)
• Others^f^	6 (6)
**Clinical outcomes**	
Length of hospitalization (days)	
3–4	38 (38)
5–6	37 (37)
7–8	15 (15)
9–10	4 (4)
>10	7 (7)
Ventilators usage	11 (11)
Antibiotic treatment	95 (94)
Antiviral treatment	2 (2)
**Outcomes**	
Not severe	49 (49)
Pneumonia	42 (42)
Death	8 (8)
Need special care	3 (3)
Bronchiolitis	1 (1)

^a^ Some patients showed more than one symptom.

^b^ Other symptoms included sore throat (40%), myalgia (27%), rhinorrhea (21%), headache (21%), chills (20%), diarrhea (13%), vomiting (5%), and hoarseness (1%).

^c^ Some patients had more than one comorbidity.

^d^ Other comorbidities included immunodeficiency (8%), cancer (4%), tuberculosis (4%), obesity (3%), leukemia (3%), brain or nerve disorder (2%), rheumatoid arthritis (2%), asthma (1%), epilepsy (1%), multiple myeloma (1%), systemic lupus erythematosus (1%), and thalassemia (1%).

^e^ Some patients exhibited more than one abnormal chest X-ray.

^f^ Other abnormal chest X-rays included lung cavity (2%), cardiomegaly (1%), pneumomediastinum (1%), lung mass (1%), and pulmonary congestion (1%).

### Prevalence of respiratory pathogens

**Fig 1:**
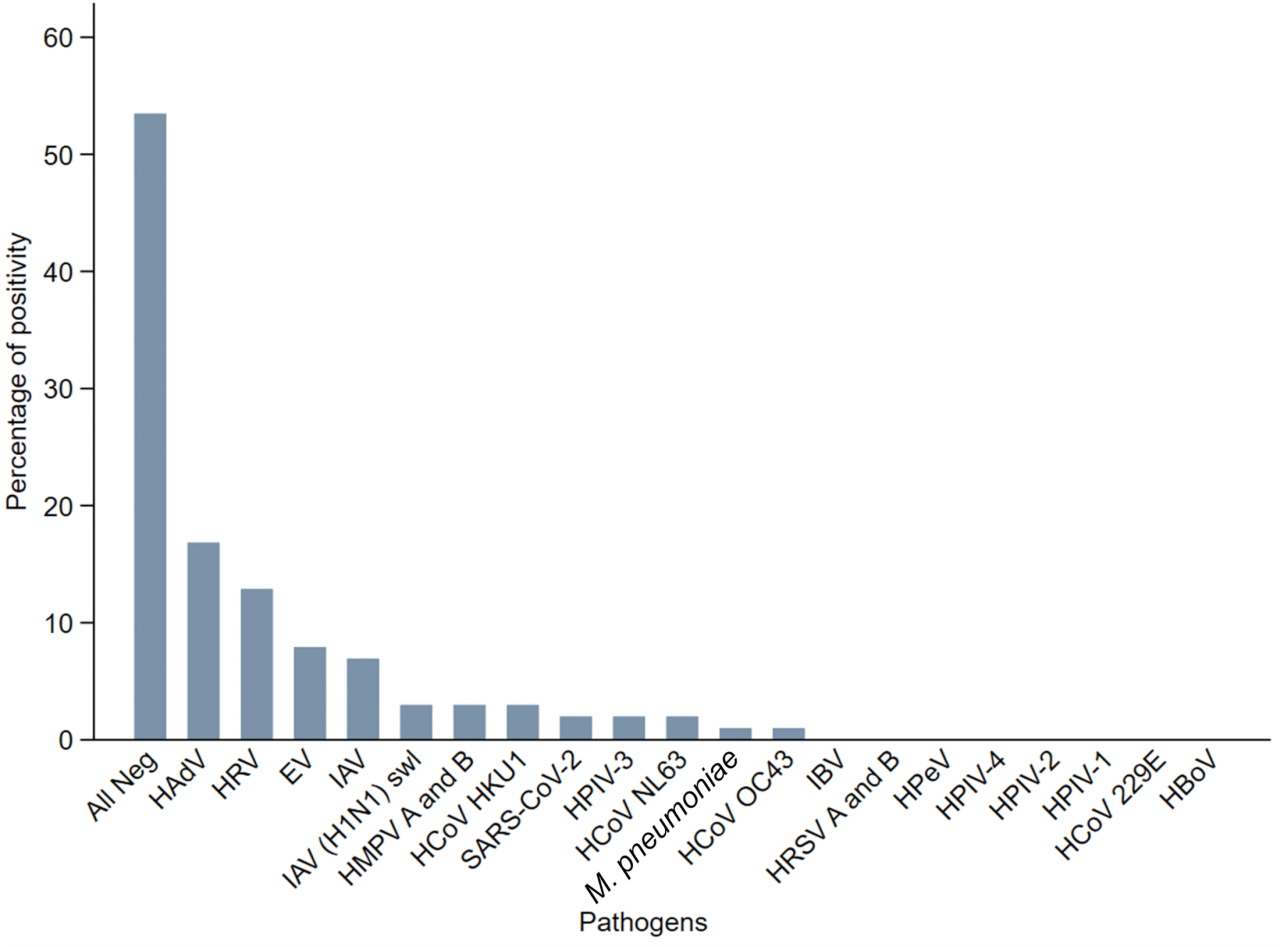
Prevalence of respiratory pathogens in patients with severe acute respiratory infections in the northern region of Thailand. The x-axis indicates the different pathogens in the 21 FTD multiplex real-time RT-PCR panel. The y-axis shows the percentage of positive results for each pathogen.

Remnant respiratory samples collected included sputum (n = 100) and nasopharyngeal swab (n = 1). Of the 101 samples tested using the FTD™ 21 multiplex RT-PCR panel, 47% were positive for at least one respiratory pathogen included in the testing panel (Fig 1). The most frequent pathogens detected were human adenovirus (17%) and human rhinovirus (13%). Other detected viruses included enterovirus (8%), influenza A virus (7%), human metapneumovirus (3%), and coronaviruses NL63, HKU1, and OC43 (combined 6%). Of the 7% influenza A virus, 3% was found to be the 2009-pandemic swine-lineage influenza A virus subtype H1N1 (IAV [H1N1] swl). One case of *Mycoplasma pneumoniae* was identified, and 53% of samples were negative for all tested pathogens.

### Mono- and co-infection of respiratory pathogens

**Fig 2:**
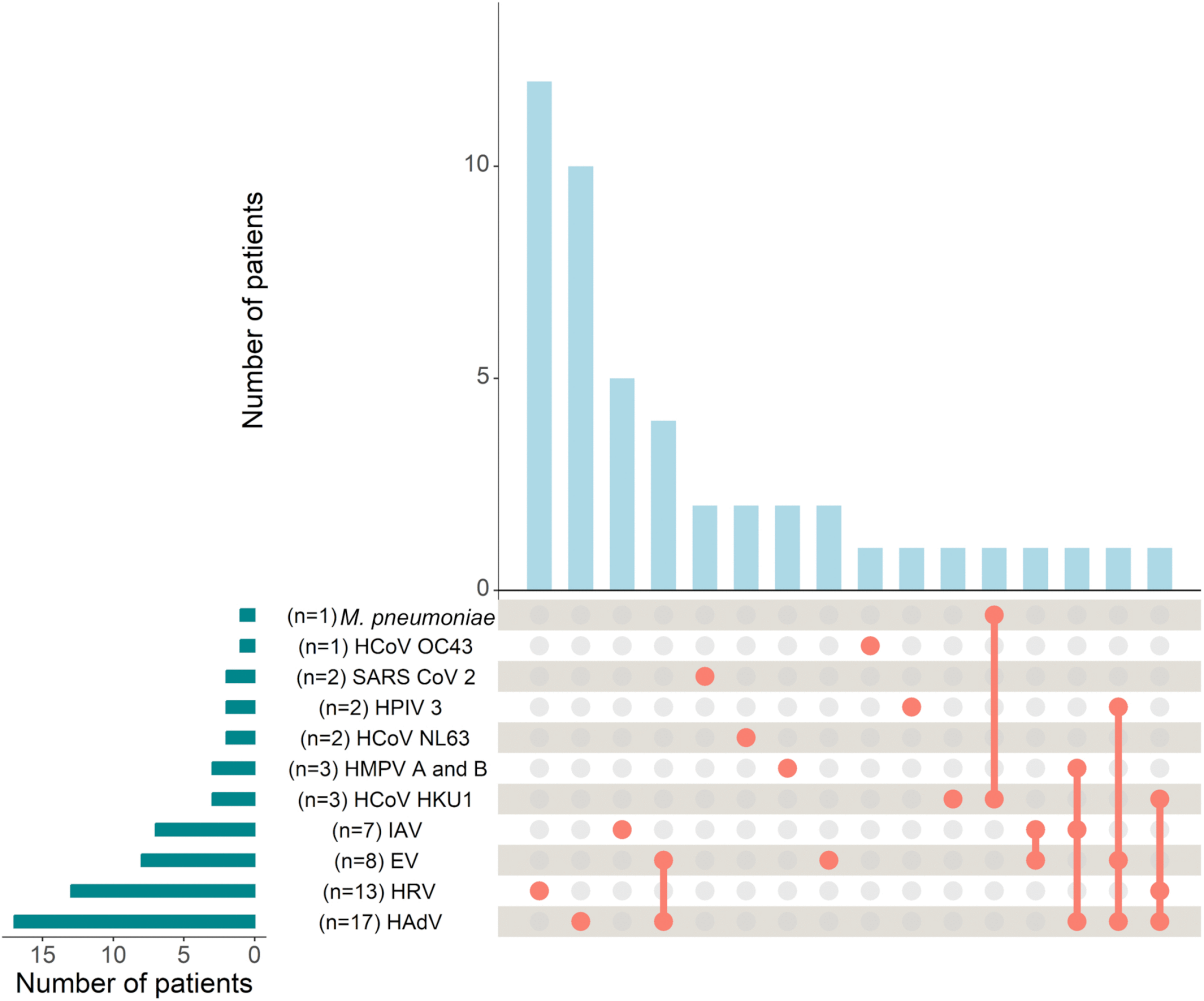
Illustration of patterns of mono- or co-infection with at least 2 or more pathogens in SARI patients. The horizontal bar plot on the left shows the total number of patients in whom each respiratory pathogen was detected. The bar plot at the top showed the number of patients in whom each mono- or co-infection was found. The matrix shows the mono- or co-infection of the detected pathogens. Each dot represents a specific pathogen, and the vertical lines connecting the dots indicate co-detections in the same patient.

Co-infections with two or more pathogens were observed in 9% (9/101) of patients (Fig 2). The most common co-infection involved HAdV and EV (44%, 4/9). Other observed combinations included HAdV with IAV, HMPV, HPIV-3, HRV, and HCoV HKU1. One patient had a viral-bacterial co-infection (HCoV HKU1 and *M. pneumoniae*). Three cases (33%, 3/9) had triple infections.

### Relationship between pathogens and clinical outcomes

**Fig 3:**
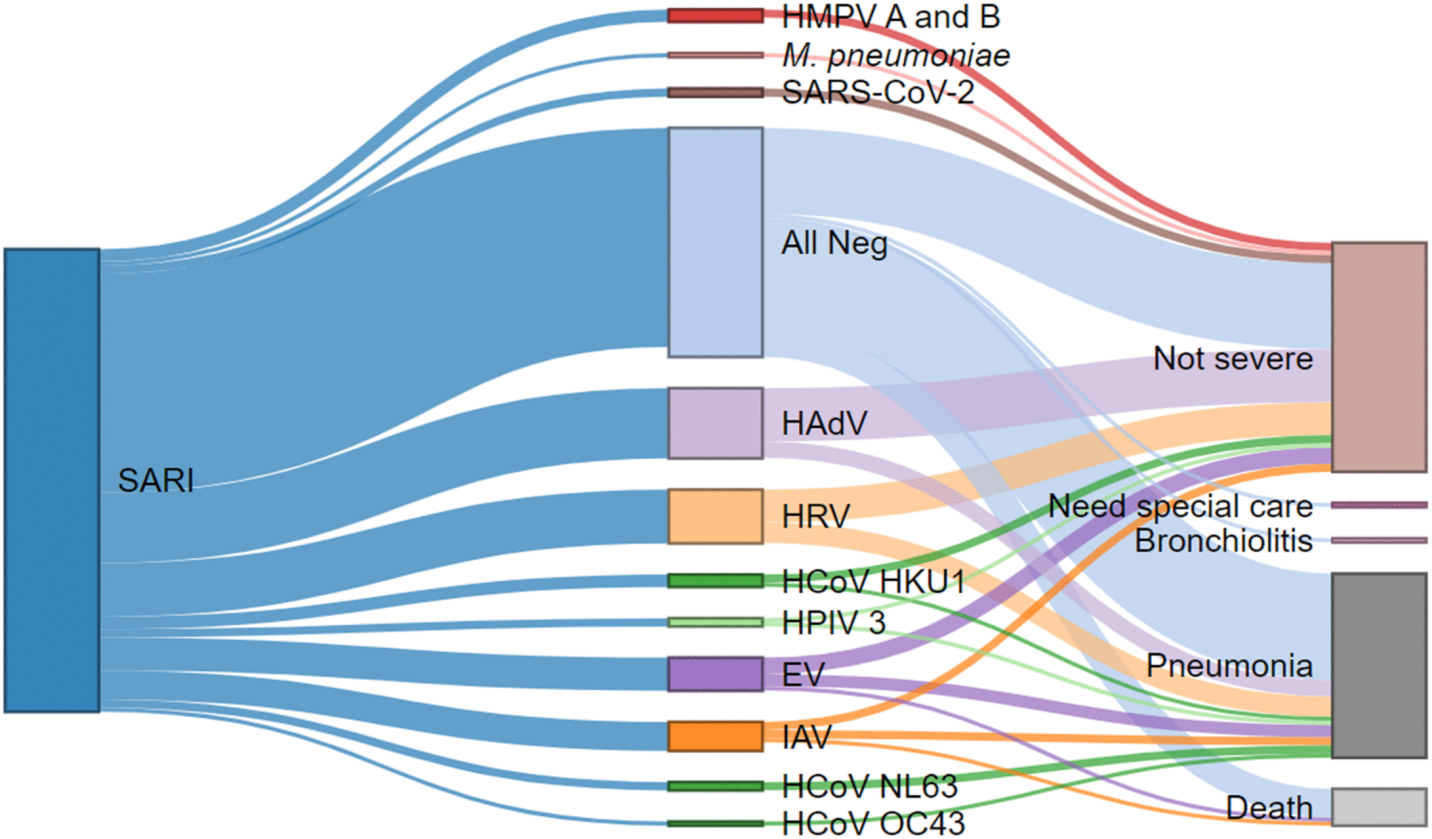
Sankey diagram showing the relationship between respiratory pathogens and clinical outcomes in SARI patients. The leftmost node represented all SARI patients included in this study. The middle layer categorized patients by the pathogen detected through multiplex PCR testing. All Neg denoted patients in whom no respiratory pathogen was detected. The rightmost node indicates clinical outcomes from the hospital database, which include not severe, need special care, bronchiolitis, pneumonia, and death. The width of each flow is proportional to the percentage of patients in each category.

To explore the relationship between detected pathogen and clinical outcomes, we examined outcomes of the patients with either mono-infections or co-infections. Patients with mono-infections more frequently had shorter hospital stays (<4 days; 19/38, 50%), whereas 5/9 (56%) of co-infected patients stayed >5 days. Co-infected patients were more often diagnosed with pneumonia (2/9, 22%) or experienced fatal outcomes (1/9, 11%), while 24/38 (63%) of mono-infected patients had no severe outcomes. Despite these observed differences, there were no statistically significant associations between infection type and clinical outcomes (*p* > 0.05). Across all positive samples, pneumonia cases (42/101, 42%) were most often associated with HRV (12/101, 12%), HAdV (10/101, 10%), and EV (7/101, 7%), as shown in Fig 3. One death occurred in a patient co-infected with IAV and EV. Several severe cases, including pneumonia and deaths, were PCR-negative, suggesting possible infections with pathogens not included in the assay panel.

### Seasonality of respiratory pathogens

**Fig 4:**
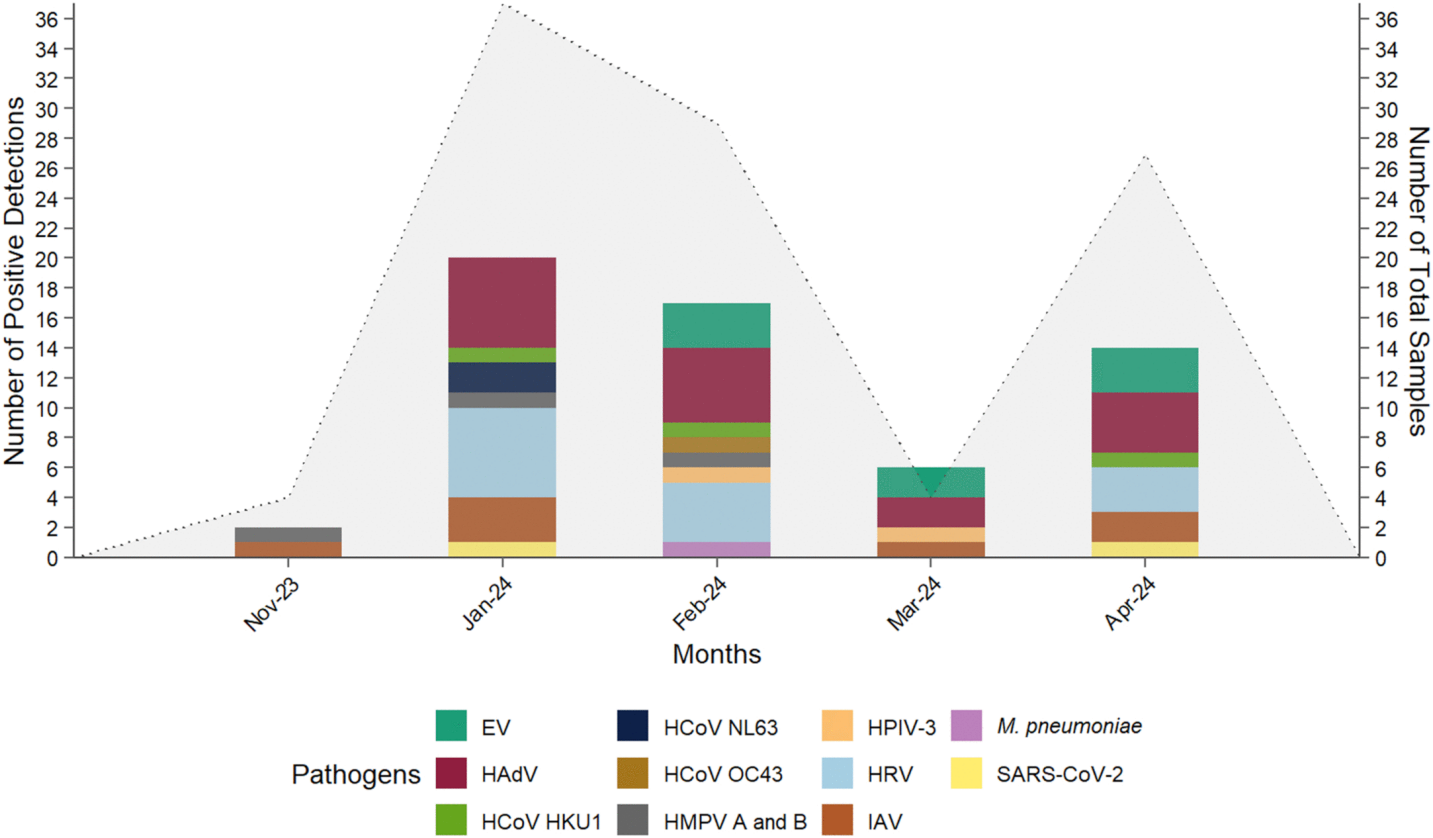
Monthly distribution of respiratory pathogens detected among adult SARI patients in northern Thailand. The stacked bars represent the number of positive detections for each pathogen. Colors correspond to specific pathogens as indicated in the legend. The dotted line indicates the total number of samples tested per month. EV: Enterovirus; HAdV: Human adenovirus; HCoV: Human coronavirus; HMPV: Human metapneumovirus; HPIV-3: Human parainfluenza virus type 3; HRV: Human rhinovirus; IAV: Influenza A virus; *M. pneumoniae*: *Mycoplasma pneumoniae*; SARS-CoV-2: Severe acute respiratory syndrome coronavirus 2.

Distinct temporal patterns were observed. Influenza A virus peaked in January. HRV exhibited a winter peak from January to February, while HAdV and EV were detected throughout the surveillance period, suggesting year-round circulation. HMPV cases were sporadic, with detections in November, January, and February. Other viruses, such as SARS-CoV-2 and HPIV-3, were detected intermittently without a clear seasonal trend (Fig 4).

## Discussion

This retrospective analysis of 22 respiratory pathogens in adult patients with SARI, hospitalized between November 2023 and April 2024, provides critical insights into the post-COVID-19 etiological spectrum and clinical characteristics of SARI. Using multiplex PCR, we detected at least one respiratory pathogen in 47% of patients, with 9% exhibiting co-infections. The median age was 62 years, and most patients had at least one comorbidity (79%) and received empirical antibiotic therapy (94%). Pneumonia was the most common clinical complication.

Nearly half (47%) of SARI cases had a detectable respiratory pathogen within the testing panel, consistent with prior molecular diagnostic studies reporting detection rates between 30% and 70% depending on population, season, and diagnostic platform [17–19]. Adenovirus was the most frequently detected pathogen (17%), followed by rhinovirus (13%). Although adenovirus has traditionally been linked to pediatric and immunocompromised populations [20,21], recent evidence supports its role in severe adult respiratory disease [22,23] with potential for nosocomial transmission in institutional settings [24,25]. Rhinovirus, often associated with mild upper respiratory illness, is increasingly implicated in severe lower respiratory tract infections in elderly and comorbid populations [26,27], where it may trigger exacerbations of chronic pulmonary disease [28,29]. The prevalence of rhinovirus in our study mirrors an earlier report where 15% of rhinovirus was found [17].

Other pathogens included enterovirus (8%), influenza A virus (7%), and human metapneumovirus (3%). Influenza A, a well-known cause of seasonal SARI [30], showed a relatively low prevalence, possibly reflecting the combined impact of high influenza vaccination coverage (73%) and residual effects of non-pharmaceutical interventions (NPIs) implemented during the COVID-19 pandemic [31–33]. In addition, our surveillance period (November 2023 to April 2024) was outside of the primary seasonal peaks of influenza in Thailand, which usually occur during the rainy season (August–September) and the winter season (January–February) [34]. This timing may have contributed to the relatively low detection rate, particularly after the early-season peak in January. Endemic coronaviruses were detected in 6% of cases, while SARS-CoV-2 was rarely found, likely due to high vaccination uptake and waning pandemic waves. Only one case of *Mycoplasma pneumoniae* was detected, which may reflect both the assay’s limited bacterial target range and recent shifts in respiratory pathogen circulation [2,16]. However, a bacterial infection other than *M. pneumoniae* cannot be ruled out among the 53% of cases who were tested PCR negative in this study.

Co-infections were identified in 9% of cases, primarily viral–viral combinations. Statistical analysis revealed no significant differences in hospital stay duration or severe clinical outcomes between patients with mono- and co-infections. These findings contrast with previous studies, which have reported that co-infections are associated with longer hospital stays and more severe outcomes [35,36]. The observed discrepancy may be attributable to variations in study population characteristics, regional epidemiological factors, or differences in healthcare management practices across study settings. Older age and comorbidities were common in our cohort (median age 62 years; 45% ≥ 65 years; 78% with ≥1 comorbidity) and were strongly associated with worse clinical outcomes, consistent with global SARI data [37]. Over half of patients had abnormal chest radiographs, highlighting the diagnostic challenge of differentiating viral from bacterial pneumonia based on clinical and radiological findings alone.

A particularly notable finding was the very high rate of empirical antibiotic use (94%) despite the predominance of viral pathogens, reflecting global trends in SARI management and underscoring the urgent need to strengthen antimicrobial stewardship [38]. In contrast, antiviral use was extremely low (2%), likely due to diagnostic delays and limited access to specific agents. Most patients had short hospital stays (3–6 days), 11% required mechanical ventilation, and overall mortality was 8%, comparable to other adult SARI cohorts worldwide [39,40].

The COVID-19 pandemic has profoundly altered respiratory virus epidemiology. NPIs such as mask-wearing, physical distancing, and travel restrictions sharply reduced seasonal respiratory virus activity [41]. As these measures were lifted, viral circulation resumed asynchronously, with rhinovirus re-emerging earliest, followed by adenovirus and others [42]. The low detection of SARS-CoV-2 and influenza in this study may reflect continued vaccine protection and residual NPI effects.

Our findings have several implications for SARI surveillance and management in Thailand and similar settings: 1) Enhanced surveillance using molecular diagnostics is essential to monitor the evolving respiratory virus landscape in the post-pandemic era; 2) Targeted vaccination strategies should be maintained and expanded for high-risk populations; 3) Antimicrobial stewardship must be strengthened through rapid diagnostics and clinical decision support to reduce unnecessary antibiotic use; 4) Improved clinical management requires timely access to pathogen-specific antivirals and broader diagnostic coverage; 5) Preparedness for emerging threats demands robust laboratory capacity, integrated surveillance systems, and efficient data sharing.

This study has limitations. First, it was conducted at a single tertiary hospital, which may limit generalizability. The study site is not part of Thailand’s official SARI sentinel surveillance network coordinated by the Ministry of Public Health or WHO. This study, therefore, represents an independent, hospital-based initiative. While the findings may not be generalizable nationwide, these offer valuable local insights and highlight the need to strengthen decentralized surveillance systems. Expanding surveillance to multiple sites would help capture a broader and more representative patient population. Second, the use of remnant clinical specimens introduces potential selection bias, as only patients who underwent respiratory testing were included. A prospective study with sampling of all eligible SARI patients could reduce this bias. Third, the multiplex PCR panel used did not cover all bacterial and viral pathogens, potentially underestimating the full etiological spectrum. In the future study, increasing pathogen coverage using next-generation sequencing or metagenomics could provide a more comprehensive assessment. Lately, retrospective data collection limited the availability of some clinical details, such as symptom onset timing, prior antibiotic use, and vaccination history. Integrating clinical and epidemiological data will be critical for refining diagnostic strategies and guiding evidence-based public health interventions. Despite these limitations, this study provides valuable insight into the post-pandemic burden and etiological profile of adult SARI in northern Thailand.

## Conclusions

In this post-COVID-19 pandemic surveillance study, nearly half of hospitalized adult SARI cases in northern Thailand were associated with detectable viral pathogens, most commonly adenovirus and rhinovirus. However, bacterial infection cannot be totally excluded due to the nature of the testing panel used in our study. Co-infections, while uncommon, were linked to more severe outcomes. The limited diagnostic yield of current multiplex PCR panels, coupled with high empirical antibiotic use, underscores the urgent need for expanded diagnostics and stewardship interventions. Seasonal patterns observed in pathogen circulation can inform future surveillance and preparedness strategies. Sustained investment in molecular diagnostics, vaccination programs, and antimicrobial stewardship is critical for effective management of SARIs in the post-COVID-19 era.

## Supporting information

S1 Table Detailed demographic, clinical characteristics, and PCR results of adult patients with severe acute respiratory infection (SARI). (XLSX)
